# New support roller profile design for railway wheel re-profiling process by under-floor lathes with a single cutting tool

**DOI:** 10.1038/s41598-021-04190-y

**Published:** 2022-01-07

**Authors:** Eduardo Corral, Jesús Meneses, M. J. Gómez García, Cristina Castejón, Juan Carlos García-Prada

**Affiliations:** 1 MaqLab Research Group, Universidad Carlos III de Madrid, Leganes, Spain; 2 UNED National University of Distance Education, Madrid, Spain

**Keywords:** Mechanical engineering, Techniques and instrumentation, Civil engineering

## Abstract

The wheel re-profiling is an important part of railway wheelset maintenance. Researchers and railway operators have been very concerned about how to minimize the loss of time during wheel re-profiling without decreasing safety. Avoiding wheelset disassembly means considerable time savings, while reducing wheel damage during operation. Underfloor wheel lathes are the most appropriate tool to achieve this double objective, and therefore the most used nowadays. Multi-cut tool lathes have the disadvantage of being extremely expensive. On the other hand, with single tool lathes, re-profiling is not smooth or safe enough when current convex profile support rollers are used. It is well known by the companies that during reprofiling the wheel suffers impacts/damaged. In this article, a methodology to optimize the profile of the support rollers used in underfloor single tool lathes for railway wheel re-profiling is proposed. This novel profile design will minimize damage and increase the safety of such lathes, since it proposes a greater smoothness in the process. Simulations of re-profiling process have been carried out by the finite element method showing that the designed roller profile reduces drastically the impact/damage during the operation. The impact generated between the re-profiling wheel and the rollers is avoided. Profile-optimized support rollers have been used in a real underfloor wheel lathe, showing good results.

## Introduction

Improving the safety and security of transport has been a major objective in the last decades. Especially, the railway industry requires careful consideration for the problems related to the contact between wheel and rail.

Predictive maintenance and inspections of each rolling element of the wheelset have become vitally important. Non-traditional techniques that have been developed for general rotating machinery are starting to be applied for wheelsets condition monitoring^1^. The rolling elements are the most critical due to vibrations^2,3^.

Wheels are the most important moving parts of a train that have a crucial impact on driving safety. Due to the spreading and complexity of the contact force between the wheelset and the rails, the wheel tread and flange may lose their original shape due to the damage after certain kilometers of travel.

The crushing of railway wheels is induced by unintentional sliding between the wheel and the rail at the moment of breakage^4,5^. The impact load caused by wheel crushes depends on the depth and length of the crushes, as well as on the speed and load of the train. This impact load can be several times higher than the static wheel load^6–9^. Wheel crushes are the main causes of wheel bearing damage, increased axle temperature, axle fracture, as well as rail and concrete sleeper fracture^10,11^.

These problems, in particular wheel wear, are particularly important in the railway industry. The most critical wheel wear problems is the "rail wheel flats". The reason for the appearance of these “Railway wheel flats” is the generation of an unintentional slippage between the wheel and the rail^4,5^. This wear generates impact loads that depend on the depth and length of the impact, as well as on the train load and train speed. This impact load can be several times higher than the wheel static load^6–9^. Wheel flats are the main causes of wheel bearing damage, axle temperature rise, axle fracture, as well as rail and concrete sleeper fracture^10,11^.

When the wheel does not have the correct profile due to wear; the quality, comfort, stability and safety of the train is diminished, as well as the ultimate life of the rails^12–14^.

For these reasons, the wheels have to be re-profiled to restore its geometry tread for purpose of maintenance. The maintenance and safety testing of wheels, in particular the reprofiling operation, is critical for the railway industry. A lot of research is currently being carried out to improve wheel maintenance.

Nowadays, every year tens of millions of railway wheel are re-profiled.

The profiling and re-profiling is a subject of current research with several methods, like the roller burnishing^15^. These methods are being optimized more and more^16^. These methods are complex and depend on many factors, such as the position of the cutting tool^17^, of the verification of its curvature^18^, or the importance of the surface quality^19^. Hoon Hun applied an optimization of a roller levelling with finite element analysis^20^. Yanglin Peng developed a method to control the tool deflection error^21^.

**Figure 1:**
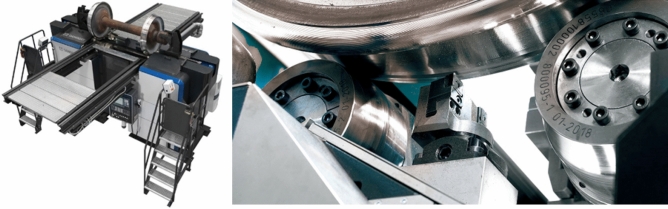
Single tool underfloor wheel lathe for railway wheel re-profiling.

Currently, the re-profiling process is commonly carried out by turning and mold-milling. For example, the Danobat under wheel lathe (see Fig. 1) is a specific machine tool for the corrective maintenance of railway rolling surfaces. It can perform the re-profiling without the need for dismounting the train axle and is equipped with the latest technology. The design performed in this study has been applied to a under wheel lathe of this type and has shown good results. The Stanray TM Underfloor Wheel Truing Machine (TN-84C)^22^ is an underfloor pit-mounted milling machine capable of simultaneously re-profiling both wheels of a railway wheel set using the axle centers as the machining reference point.

At the moment, there are not many studies on re-profiling in the literature. The industry is demanding methods to optimize reprofiling. Zhang et al. through multi-target optimization propose a reprofiling that reduces the amount of material removed. This improves yield and service life without reducing safety^23^. Chien et al. designed a predictive model of re-profiling period by statistical and mathematical methods^24^. As for the tread re-profiling process, there are not many scholars in the literature. Seo et al. studied fatigue and the effects of metal removal on wheels using finite elements^25^. Filipowicz et al. used a finite element computer model to test the feasibility of turning resilient wheels on a lathe. Resilient wheels reduce noise and improve traveler comfort^26^. Tian made a theoretical basis for the selection of tools for re-profiling^27^. Cioboata et al. made a study of profiling/reprofiling and how to measure railway wheels^28^. Andrade and Stow performed a sensitivity analysis on underfloor wheel lathes, using stochastic frontier analysis, and considering several variables such as flange thickness, fatigue and wheel flats^29^.

In summary, researchers have so far made a great deal of research and got lots of achievements regarding wheel profile optimization, machined surface, cutting heat and cutting force, and so forth during metal cutting. However, after extensive consultation, the authors have not found literature on the optimal shape of the support rollers used in underfloor lathes for railway wheel re-profiling.

This paper proposes a method to find the optimal shape of the support rollers of a single tool underfloor wheel lathe, to avoid sudden load transfers during the process. The new shape of the roller profile has been analyzed through the dynamic simulation analysis of the wheel re-profiling process by means of a finite element method.

## Description of the problem associated with a single tool under-floor lathe for railway wheel re-profiling

In this section, the problem associated with a single tool underfloor lathe equipped with support rollers (two rollers for each wheel, at least one of them motorized) is described.

**Figure 2:**
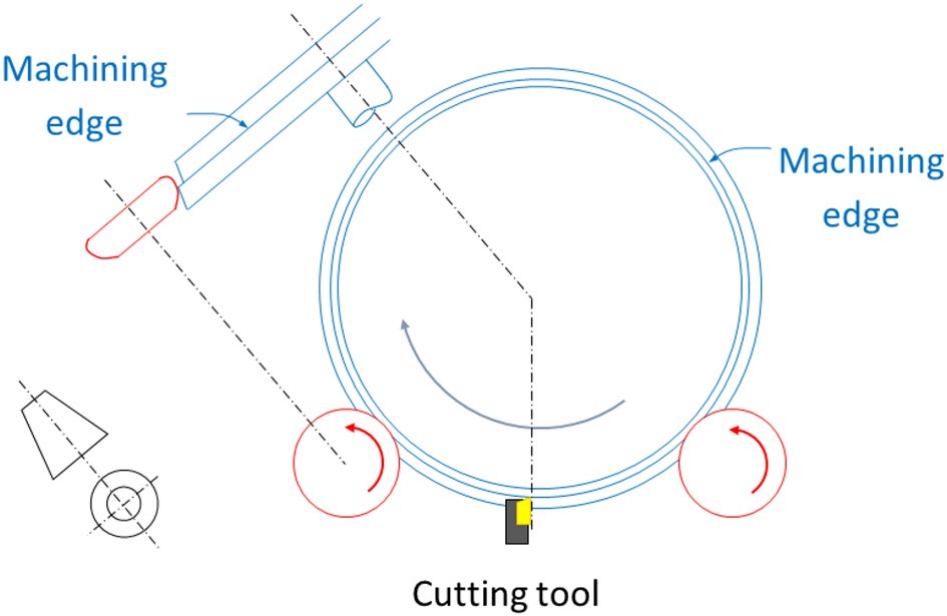
Front and side views showing the configuration of the support rollers (red), the cutting tool (yellow) and the wheel to be re-profiled (blue). The machining edge during the re-profiling process is also shown.

The cutting tool works in a vertical plane between both rollers (see Fig. 2).

**Figure 3:**
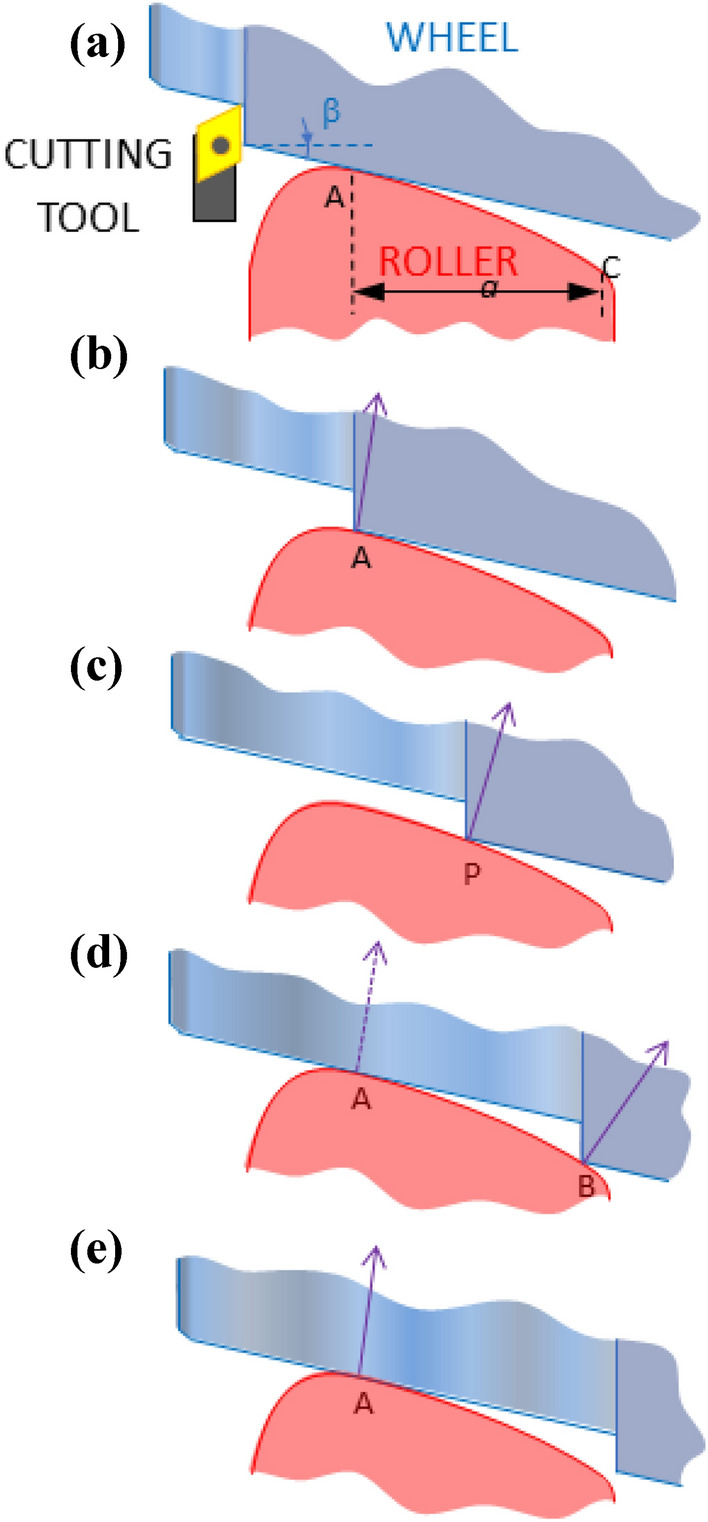
Cross section of roller and wheel at different re-profiling phases (the cutting tool drawn in a) is out of section).

Nowadays in this kind of lathes, support rollers are used whose profile is an arc of circumference, so they are totally convex. To illustrate the problems associated with these roller profiles, in Fig. 3 several phases of the re-profiling process of a wheel (in blue), of conicity semi-angle − *β*, supported on a generic profile roller (in red) are presented. The slope of the roller profile will be negative in the area of the roller that we will call “useful” (AC section), of width *a*.

At the beginning, the wheel rests on the roller at point A, where the wheel slope and the roller slope are both equal to *β* (Fig. 3a). This point will be the support point until it is reached by the machining edge (Fig. 3b). From then on, the support will take place on the machining edge (point P), which runs along the profile of the roller (Fig. 3c). Meanwhile, the already re-profiled surface will approach the point of the first roller support, point A. When the machining edge reaches point B, a support transfer occurs (Fig. 3d), at the end of which, the wheel will be supported again on point A, on its already re-profiled area (Fig. 3e).

Throughout the re-profiling process, the contact force between the roller and the wheel changes in its direction and in its distribution over the contact area(s). In fact, a load transfer from contact on point B to contact on point A occurs in phase (d) (Fig. 3). This load transfer supposes an abrupt decrease in its axial component, and usually causes undesirable impact and stability problems, so it is worth studying it in depth. If we consider the roller and wheel as rigid solids, the load transfer between B and A would be instantaneous; but if they are considered as deformable bodies, the contact surface at B will decrease while the contact surface in A will increase, resulting in a less abrupt load transfer.

The new design of the roller profile is first optimized considering the roller and the wheel as rigid solids. Once the optimal profile is proposed, the process will be studied considering both as deformable solids. For this purpose, the finite element technique will be used.

## Roller profile design

In the intermediate phase of the re-profiling process, when the roller-wheel contact occurs on the machining edge, point P (Fig. 3), the already re-profiled surface of the wheel approaches towards point A of the roller, where it will eventually be supported again.

**Figure 4:**
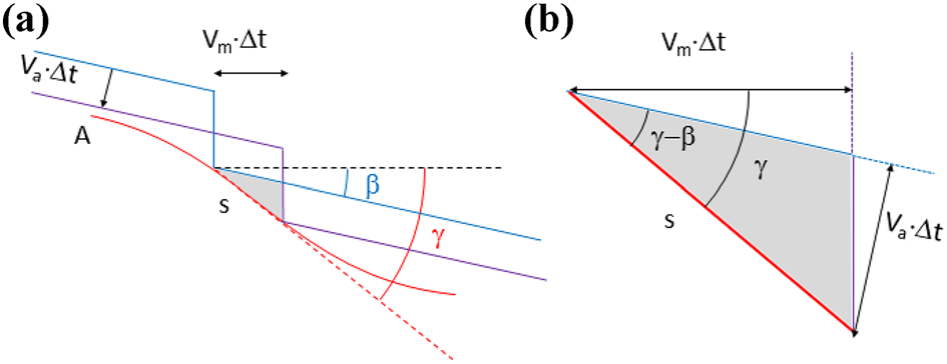
(**a**) Two successive positions (separated by the time Interval Δt) of the wheel being re-profiling (in blue and purple, respectively) supported on the roller (in red). (**b**) detail of the shaded area showing the involved velocities (machining velocity, V_m_ ; and approach velocity, V_a_) and angles (slope of the wheel, *β*; and that of the roller profile at the contact, γ).

In Fig. 4 two moments of the re-profiling process separated by a small-time interval, *Δt* (in blue and purple, respectively) have been represented. *V*_*a*_ and *V*_*m*_ are the approach and machining speeds respectively, *s* is the distance between contact points at *t* and *t* + *Δt*. It can be seen that the greater the difference between the inclination of the wheel (taper half-angle), *β* and the slope of the roller profile in the contact, *γ*, the greater the speed with which the re-profiled surface approaches to point A.

In fact, from Fig. 4 we have:

1 $$ {\text{V}}_{a} = {\text{V}}_{m} \frac{{\sin \left( {\gamma - \beta } \right)}}{\cos \gamma } $$

In rollers whose useful profile is totally convex, as those used at present, the slope of the roller is a monotonous diminishing function, reason why it goes away of the value of the inclination of the wheel as the machining edge moves away from point A. Therefore, the approach speed increases as the re-profiling process progresses, and can become considerably high at the time just before the double support (phase d in Fig. 3), producing a more abrupt load transfer than desired.

Note that for rollers with a totally convex profile, the load transfer between points A and B is accompanied by a discontinuity in the axial component of the roller-wheel contact force: in B it is much larger than in A (see Fig. 3).

For the load transfer to be smooth enough, the slope of the roller profile at point B should be only slightly higher than the wheel inclination (which coincides with the slope of the roller at point A, the flank of the wheel considered linear). Thus, the curve proposed as the roller useful profile has an inflection point, where it becomes from convex to concave. The designed profile must also ensure that the second support is made actually at point A, before:

(i) The machining edge leaves the useful area of the profile, and
(ii) The support is produced at a point other than A, on a not yet re-profiled area (undesirable support).

**Figure 5:**
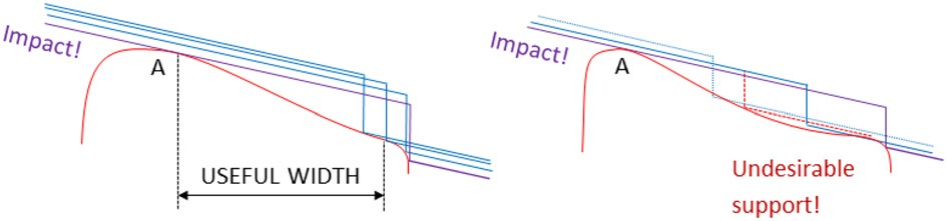
Situations that lead to a second impulsive support of the wheel in the re-profiling process: (i) the re-profiling edge leaves the area of useful width prematurely (left); (ii) there is prior undesirable support (right).

These two undesirable circumstances, represented in Fig. 5, would both imply impulsive support at point A.

**Figure 6:**
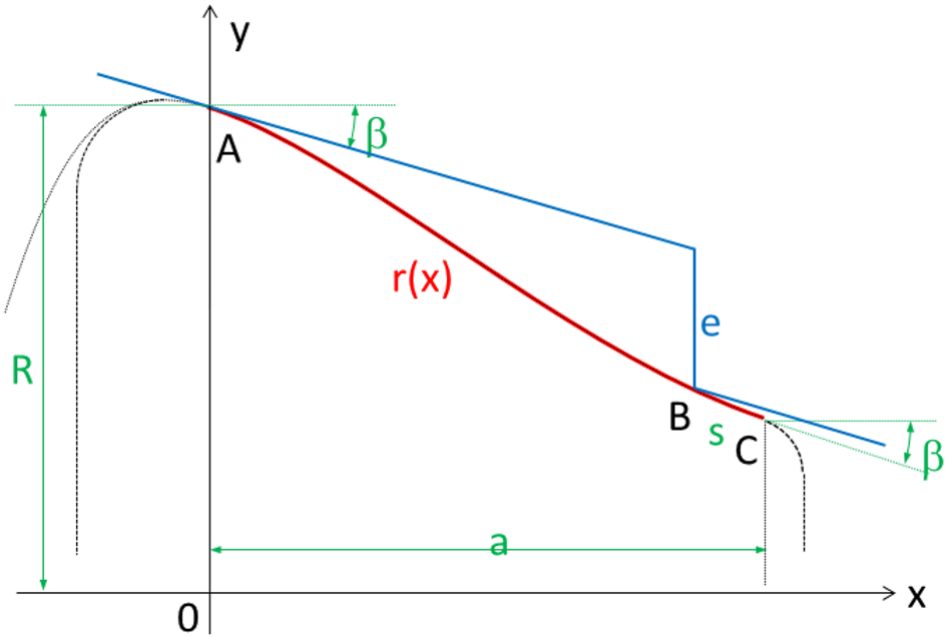
Roller profile according to constraints. Wheel profile in the double support phase of the re-profiling process. Design parameters: *β*, a, R, e, s

In particular, the profile of the roller will be described by its radius *r*(*x*), as a function of the axial distance *x*. The slope of this function should be *β* at the ends of the useful width. The parameters to consider in the profile design are: the roller radius *R* at point A, the useful width *a*, the machining depth *e*, and the slope of the wheel to be re-profiled *β*. As a design requirement, a safety section of length *s*, must also be contemplated to guarantee a desirable support before circumstance (i). This length should not be too large, so that the slope of the roller at the point B (machining edge position at the double support phase), is only slightly greater than that of the wheel. Therefore, the following constraints must be met for *r*(*x*) (see Fig. 6):

1. *r*(0) = *R*, roller radius.
2. *r*’(0) = *r*’(*a*) = – tan *β*
3. The load transfer occurs when the machining edge is located at point B, at the safety section *s*, from the last point of the useful area, C.

Limitations (i), (ii) and constraint (C2) impose an essential geometrical feature to the profile of the roller: it must be composed of a convex section followed by a concave section; therefore, it should have an inflection point in the useful area. The simplest and easiest-to-manufacture roller profile is one composed of two tangent circumference arcs. Below is the calculation process to obtain the optimal profile of this type of roller.

Optimal roller profile calculation involves the resolution of a nonlinear system of equations. For that reason, the proposed profile has been calculated in two steps. In the first one, no safety section is considered, which leads to a system of equations that can be solved exactly; the corresponding results are utilized in the second step, as the initial guess for the Newton–Raphson’s iterative method.

### First step: profile calculation without considering safety section

**Figure 7:**
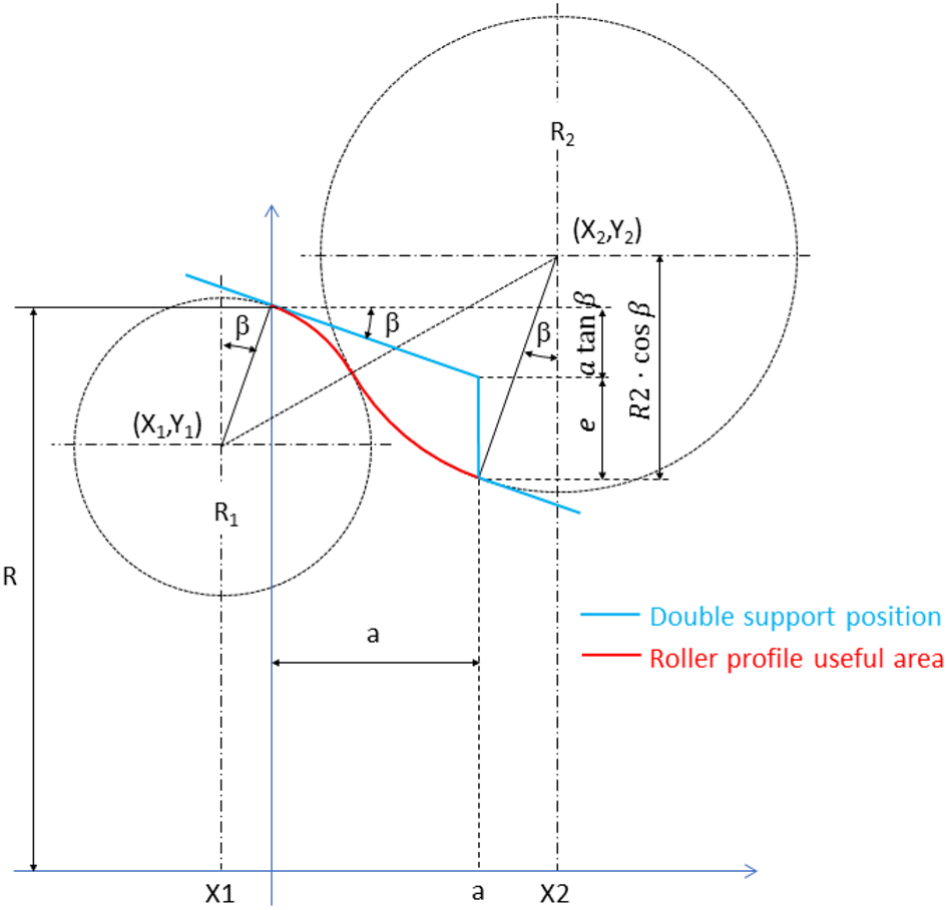
Roller profile composed of two circumference sections. Positions of the circumference centres and radios. First approach design without considering safety distance.

The Fig. 7 shows the roller profile composed of two circumference sections, one convex and one concave, having an inflection point between them. The position of the wheel profile is also shown at the moment of re-profiling process in which double support occurs. As can be seen, at that moment the machining edge reaches exactly the right useful limit of the roller profile, no safety section being considered.

In this first approach, the design parameters are: the wheel slope, *β*; the useful area length of the roller, *a*; the machining depth, *e*; and the roller radius, *R* (corresponding to the first and last roller-wheel contact points). Whereas the design variables are the radius of arc sections, R_1_ and R_2_ and the position coordinates for the corresponding centres (X_1_,Y_1_) and (X_2_,Y_2_). These variables must fulfil the following system of equations (see Fig. 7):7

2 $$ \left\{ {\begin{array}{*{20}c} {{\text{X}}_{1} = - {\text{R}}_{1} \sin \beta } \\ {{\text{Y}}_{1} = R - {\text{R}}_{1} \cos \beta } \\ {{\text{X}}_{2} = a + {\text{R}}_{2} \sin \beta } \\ {{\text{Y}}_{2} = R + {\text{R}}_{2} \cos \beta - a\cdot\tan \beta - e} \\ {\left( {{\text{X}}_{2} - {\text{X}}_{1} } \right)^{2} + \left( {{\text{Y}}_{2} - {\text{Y}}_{1} } \right)^{2} = \left( {{\text{R}}_{1} + {\text{R}}_{2} } \right)^{2} } \\ \end{array} } \right. $$

There are 5 equations for 6 variables, so just one variable is independent. The radius of one of the arc sections (R_1_, for example) can be taken as the independent variable, the rest of variables being expressed as function of R_1_. The system is not linear but it can be solved exactly. Actually, replacing the first 4 equations in the last one and operating, we get:

3 $$ {\text{R}}_{2} = \frac{{{\text{a}}^{2} + \left( {{\text{a}}\cdot\tan \beta + {\text{e}}} \right)^{2} }}{{2{\text{e}}}} - {\text{R}}_{1} $$

The value for the rest of the variables is then obtained immediately.

If the profile is required to have one convex section followed by a concave one (as in Fig. 7), the value chosen for $R_{1}$ cannot be arbitrarily large, since the sum of radii $R_{1} + R_{2}$ has a fixed value for a set of parameters *β*, *a*, *e*. This implies the following limit for $R_{1} :$

4 $$ {\text{R}}_{1} < \frac{{{\text{a}}^{2} + \left( {{\text{a}}\cdot\tan \beta + {\text{e}}} \right)^{2} }}{{2{\text{e}}}} $$

Otherwise, the value of $R_{2}$ would be negative and the useful profile area would no longer have a concave section. Of course, a negative value of $R_{1}$ implies that the entire useful profile is concave.

In theory, it is desirable a large value of the concave arc section, so that the double support occurs more smoothly. However, the convex arc section should be sufficiently large to ensure a sturdy and stable support, since the wheel will rest on that area during most of the re-profiling process. Furthermore, the smaller the radius of a section, the greater the variation of the axial component of the roller-wheel force during the passage of the machining edge through that section.

### Second step: profile calculation considering safety section

**Figure 8:**
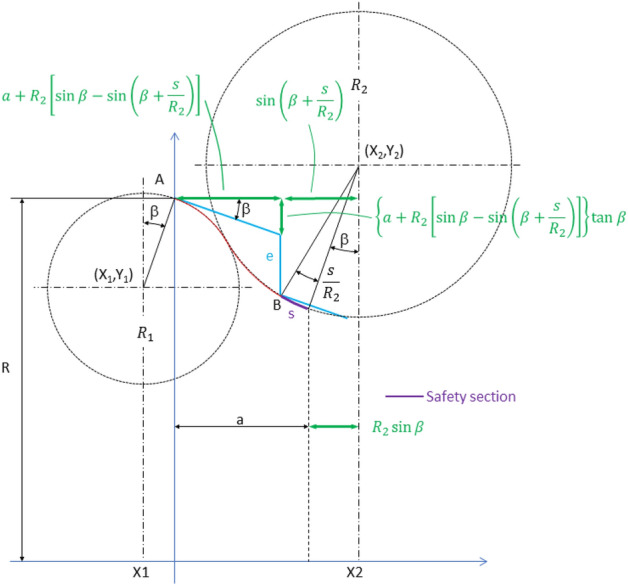
Design parameters of the roller composed of two circumference arcs, considering a safety distance, s.

When the safety section length *s*, measured along the concave section (magenta arc in Fig. 8), is considered as a design parameter, the system of equations becomes:

5 $$ \left\{ {\begin{array}{*{20}c} {{\text{X}}_{1} + {\text{R}}_{1} \sin \beta = 0} \\ {{\text{Y}}_{1} + {\text{R}}_{1} \cos \beta - R = 0} \\ {{\text{X}}_{2} - {\text{R}}_{2} \sin \beta - a = 0} \\ {{\text{Y}}_{2} + {\text{R}}_{2} \left\{ {\left[ {\sin \beta - \sin \left( {\beta + \frac{{\text{s}}}{{{\text{R}}_{2} }}} \right)} \right]\tan \beta - \cos \left( {\beta + \frac{{\text{s}}}{{{\text{R}}_{2} }}} \right)} \right\} + e + a\cdot\tan \beta - R = 0} \\ {\left( {{\text{X}}_{2} - {\text{X}}_{1} } \right)^{2} + \left( {{\text{Y}}_{2} - {\text{Y}}_{1} } \right)^{2} - \left( {{\text{R}}_{1} + {\text{R}}_{2} } \right)^{2} = 0} \\ \end{array} } \right. $$

Now, for a given value of R_1_, the first two equations (which are identical to those corresponding to the system of Eq. 2) directly provide the position of the centre of the convex arc (X_1_, Y_1_). To obtain the radius of the concave arc, R_2_, and the position of its centre, a non-linear system of three equations (the last three Eq. 5) must be solved. The unknown vector **q**, and the constraint vector **ϕ**(**q**) for this system are respectively:

6 $$ {\mathbf{q}} = \left( {\begin{array}{*{20}c} {{\text{x}}_{2} } \\ {{\text{y}}_{2} } \\ {{\text{R}}_{2} } \\ \end{array} } \right), $$

7 $$ \phi \left( {\mathbf{q}} \right) = \left( {\begin{array}{*{20}c} {{\text{X}}_{2} - {\text{R}}_{2} \sin \beta - {\text{a}}} \\ {{\text{Y}}_{2} + {\text{R}}_{2} \left\{ {\left[ {\sin \beta - \sin \left( {\beta + \frac{{\text{s}}}{{{\text{R}}_{2} }}} \right)} \right]\tan \beta - \cos \left( {\beta + \frac{{\text{s}}}{{{\text{R}}_{2} }}} \right)} \right\} + {\text{e}} + {\text{a}}\cdot\tan \beta - {\text{R}}} \\ {\left( {{\text{X}}_{2} - {\text{X}}_{1} } \right)^{2} + \left( {{\text{Y}}_{2} - {\text{Y}}_{1} } \right)^{2} - \left( {{\text{R}}_{1} + {\text{R}}_{2} } \right)^{2} } \\ \end{array} } \right) $$

The system will be solved numerically, applying the Newton–Raphson iterative method:

8 $$ {\mathbf{q}}^{{\left( {\text{j}} \right)}} = {\mathbf{q}}^{{\left( {{\text{j}} - 1} \right)}} - \left[ {\phi_{{\mathbf{q}}} \left( {{\mathbf{q}}^{{\left( {{\text{j}} - 1} \right)}} } \right)} \right]^{ - 1} \phi \left( {{\mathbf{q}}^{{\left( {{\text{j}} - 1} \right)}} } \right) $$

using as the initial guess, the vector obtained with no safety section (Eqs. 2 and 3):

9 $$ {\mathbf{q}}^{\left( 0 \right)} = \left( {\begin{array}{*{20}c} {{\text{a}} + \left[ {\frac{{{\text{a}}^{2} + \left( {{\text{a}} \cdot \tan \beta + {\text{e}}} \right)^{2} }}{{2{\text{e}}}} - {\text{R}}_{1} } \right]\sin {\upbeta }} \\ {{\text{R}} + \left[ {\frac{{{\text{a}}^{2} + \left( {{\text{a}} \cdot \tan \beta + {\text{e}}} \right)^{2} }}{{2{\text{e}}}} - {\text{R}}_{1} } \right]\cos \beta - {\text{a}} \cdot \tan \beta - {\text{e}}} \\ {\frac{{{\text{a}}^{2} + \left( {{\text{a}} \cdot \tan \beta + {\text{e}}} \right)^{2} }}{{2{\text{e}}}} - {\text{R}}_{1} } \\ \end{array} } \right) $$

Matrix $\phi_{{\mathbf{q}}}$ in Eq. (8) is the jacobian for the constraint Eq. (7):

10 $$ \phi_{q} = \left( {\begin{array}{*{20}c} 1 & 0 & { - \sin \beta } \\ 0 & 1 & {\sin \beta \tan \beta + \left[ {\frac{s}{{R_{2} }}\cos \left( {\beta + \frac{s}{{R_{2} }}} \right) - \sin \left( {\beta + \frac{s}{{R_{2} }}} \right)} \right]\tan \beta + \frac{s}{{R_{2} }}\sin \left( {\beta + \frac{s}{{R_{2} }}} \right) - \cos \left( {\beta + \frac{s}{{R_{2} }}} \right)} \\ {2\left( {X_{2} - X_{1} } \right)} & {2\left( {Y_{2} - Y_{1} } \right)} & { - 2\left( {R_{1} + R_{2} } \right)} \\ \end{array} } \right) $$

### Calculation of the axial force/radial force ratio

**Figure 9:**
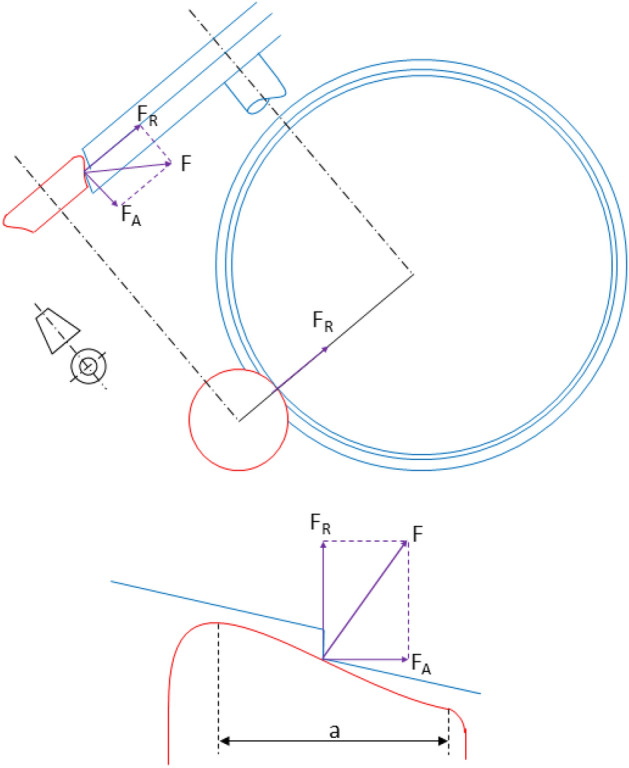
Roller-wheel contact force decomposition into radial and axial components.

In the re-profiling process, the contact (normal) force between each of the rollers and the wheel can be decomposed into a radial component, and an axial component (see Fig. 9). The contact force direction varies as the machining edge runs through the roller profile, so does the axial component to radial component ratio, $\frac{{F_{A} }}{{F_{R} }}$, that can be expressed as:

11 $$ \frac{{F_{A} }}{{F_{R} }} = \tan \varphi $$

**Figure 10:**
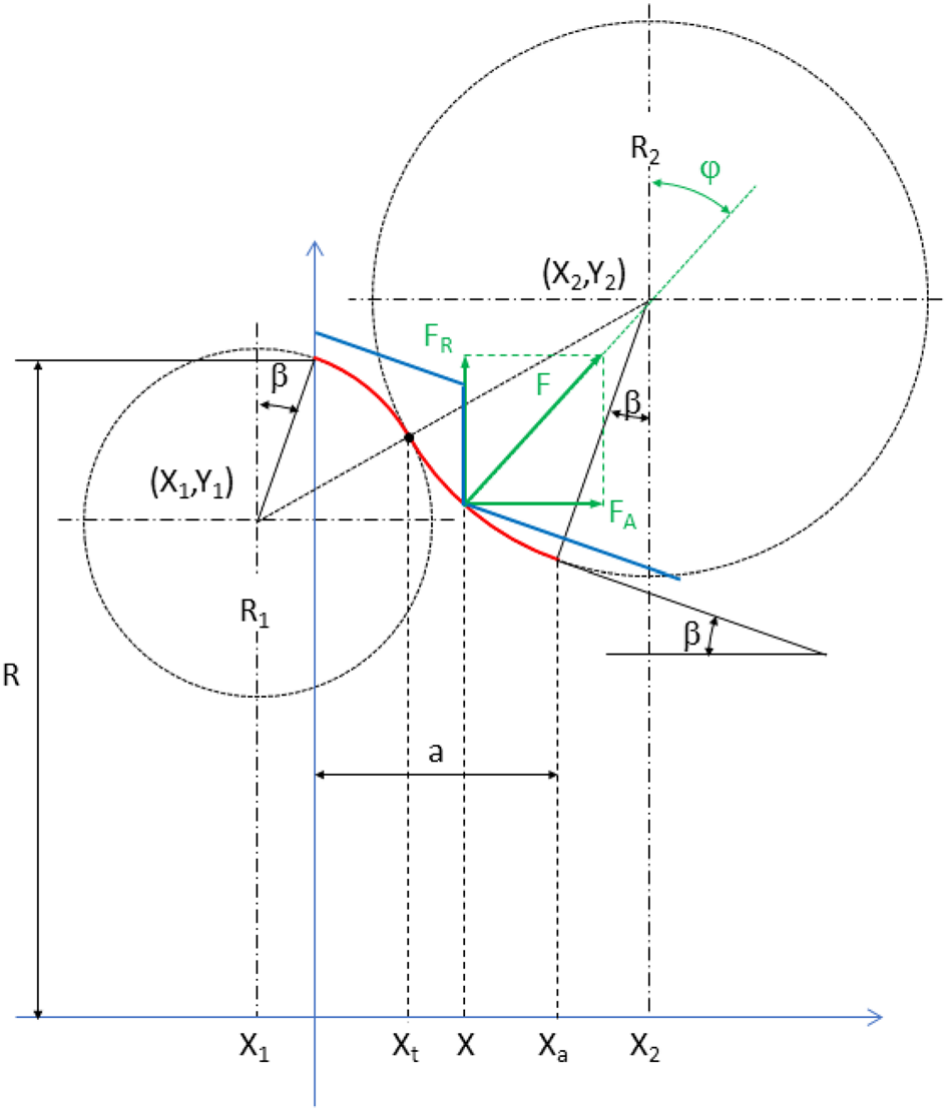
Roller-wheel contact force. Axial and radial components.

To express it in terms of the axial position X, note the relationship between X and φ (that depends on which arc section is in contact with the machining edge. See Fig. 10):

12 $$ X = \left\{ {\begin{array}{*{20}c} {R_{1} \sin \varphi + X_{1} ; 0 < X < X_{t} } \\ {X_{2} - R_{2} \sin \varphi ; X_{t} < X < X_{A} } \\ \end{array} \Rightarrow \sin \varphi = \left\{ {\begin{array}{*{20}c} {\frac{{X - X_{1} }}{{R_{1} }}; 0 < X < X_{t} } \\ {\frac{{X_{2} - X}}{{R_{2} }}; X_{t} < X < X_{A} } \\ \end{array} } \right.} \right. $$

So finally, we get:

13 $$ \frac{{F_{A} }}{{F_{R} }} = \tan \varphi = \frac{\sin \varphi }{{\sqrt {1 - \left( {\sin \varphi } \right)^{2} } }} = \left\{ {\begin{array}{*{20}c} {\frac{{X - X_{1} }}{{\sqrt {R_{1}^{2} - \left( {X - X_{1} } \right)^{2} } }}; 0 < X < X_{t} } \\ {\frac{{X_{2} - X}}{{\sqrt {R_{2}^{2} - \left( {X_{2} - X} \right)^{2} } }}; X_{t} < X < X_{a} } \\ \end{array} } \right. $$

**Figure 11:**
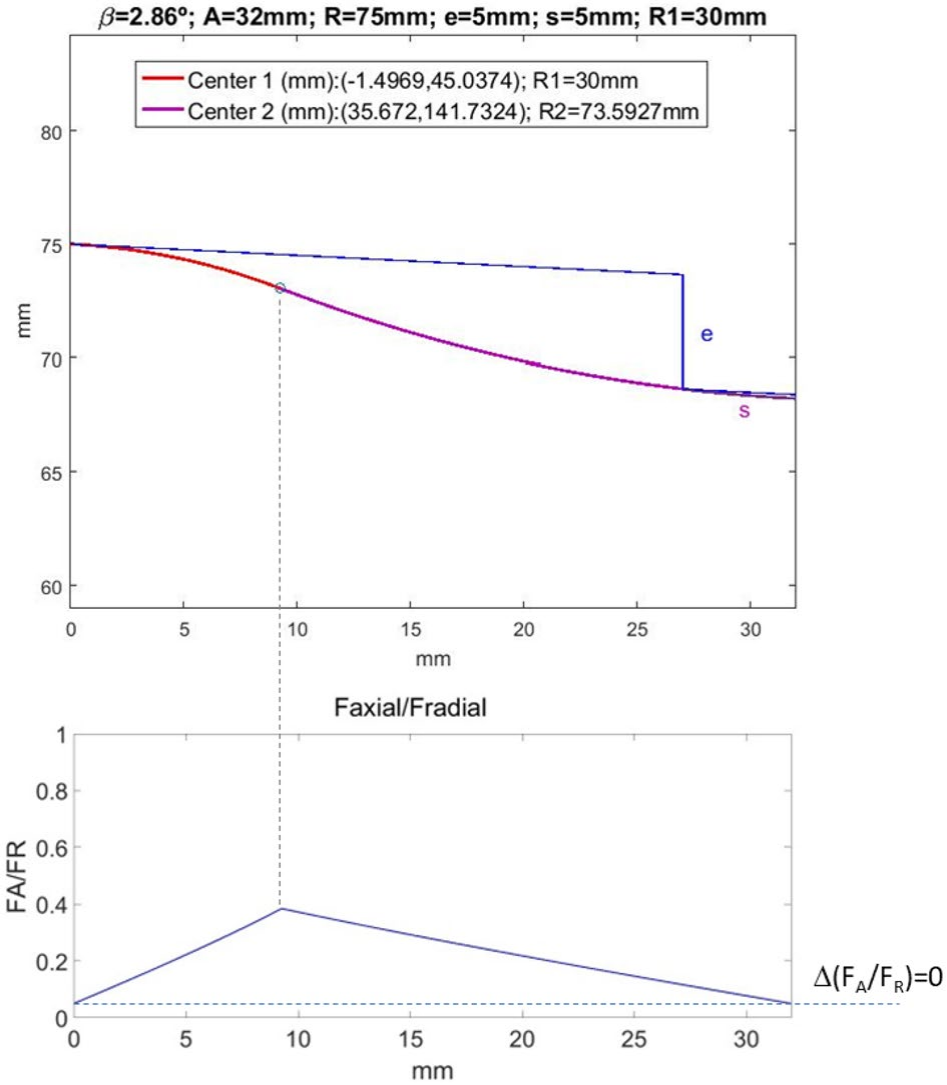
Top: optimal roller profile for the indicated design parameters. Bottom: ratio between the axial and radial components of the contact force, throughout the re-profiling process. the axial component grows to a maximum just at the inflection point of the profile. From that point on, it decreases to the same value as in the first support. As a consequence, there is no axial load transfer.

A MATLAB® code has been developed to perform the described iterative calculation and the calculations necessary to obtain the output results: X_1_, Y_1_, R_2_, X_2_, Y_2_ and F_A_/F_R_ from the input parameters: *β*, a, R, e, s, R_1_. The program also provides the graph of the roller profile together with the wheel profile in the double support position, and that of the roller-wheel contact force axial to radial components ratio. As an example, in Fig. 11 these graphics are shown for the following parameters: *β* = 2.86º, a = 32 mm, R = 75 mm, e = 5 mm, s = 5 mm, R_1_ = 30 mm. The output results are also shown.

**Figure 12:**
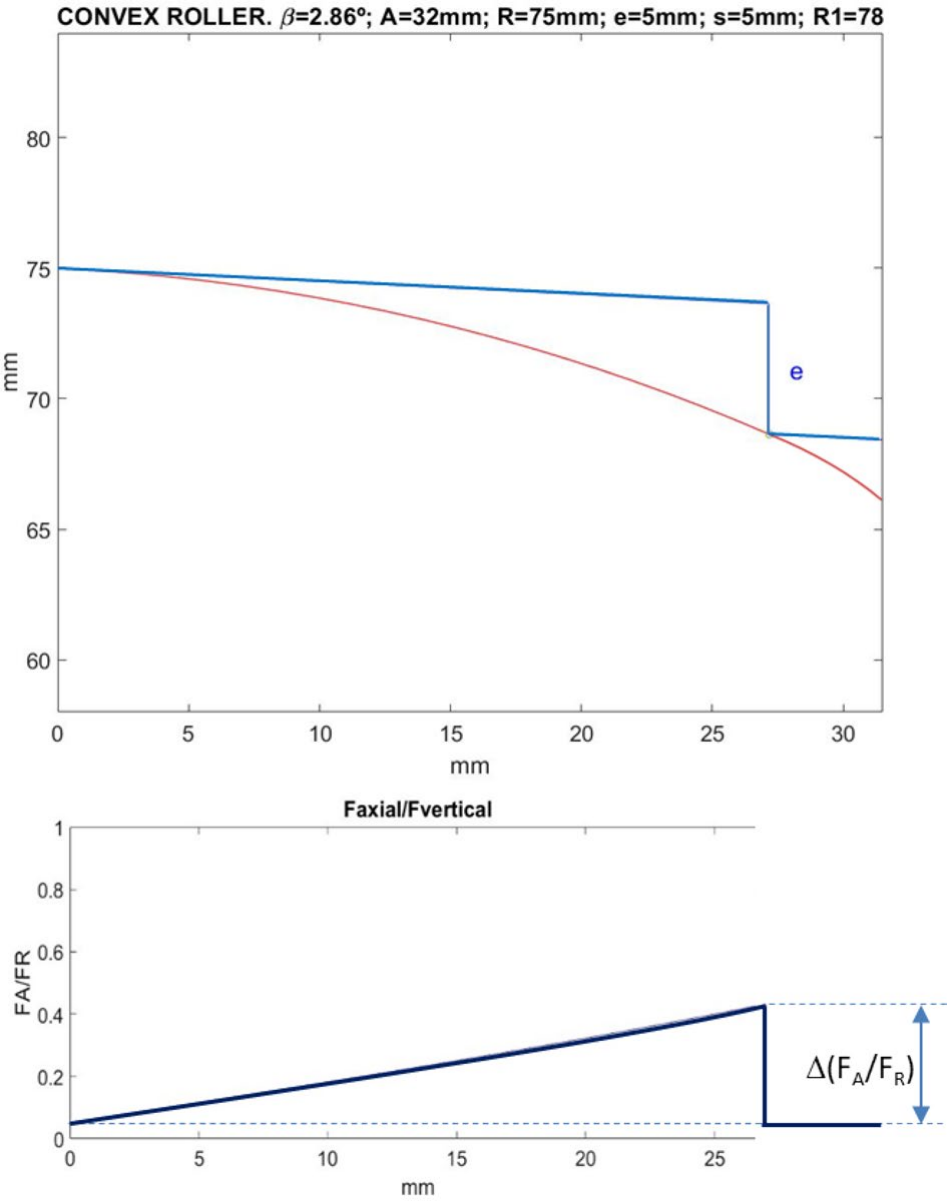
Top: Convex roller profile for the indicated design parameters. Bottom: ratio between the axial and radial components of the contact force, throughout the re-profiling process. In this case, an abrupt load transfer occurs in the second support.

Note that no practical axial load transfer occurs, as the final F_A_/F_R_ value (support at point B) practically coincides with the initial one (support at point A). On the contrary, with conventional convex rollers, the F_A_/F_R_ curve is monotonous increasing up to the point of load transfer, causing an abrupt decrease in the axial load component (see Fig. 12).

In this discussion, the cutting force exerted by the cutting tool on the wheel to be re-profiled, has been ignored. In fact, the components of the tool-to-wheel cutting force are mainly tangential and axial. Therefore, a slight reduction in the axial component of the roller-to-wheel contact force should be expected. Anyway, the axial load transfer will still occur abruptly at the “second support” when current rollers are utilized, instead of those proposed in the article, that produce no axial transfer at that moment.

## Finite element simulation of the load transfer in the double support phase

In this section, finite element results for the quasi-static load transfer process (phase d in Fig. 3) are shown. The objective is to provide a criterion for choosing the convex arc radius, R_1_.

**Figure 13:**
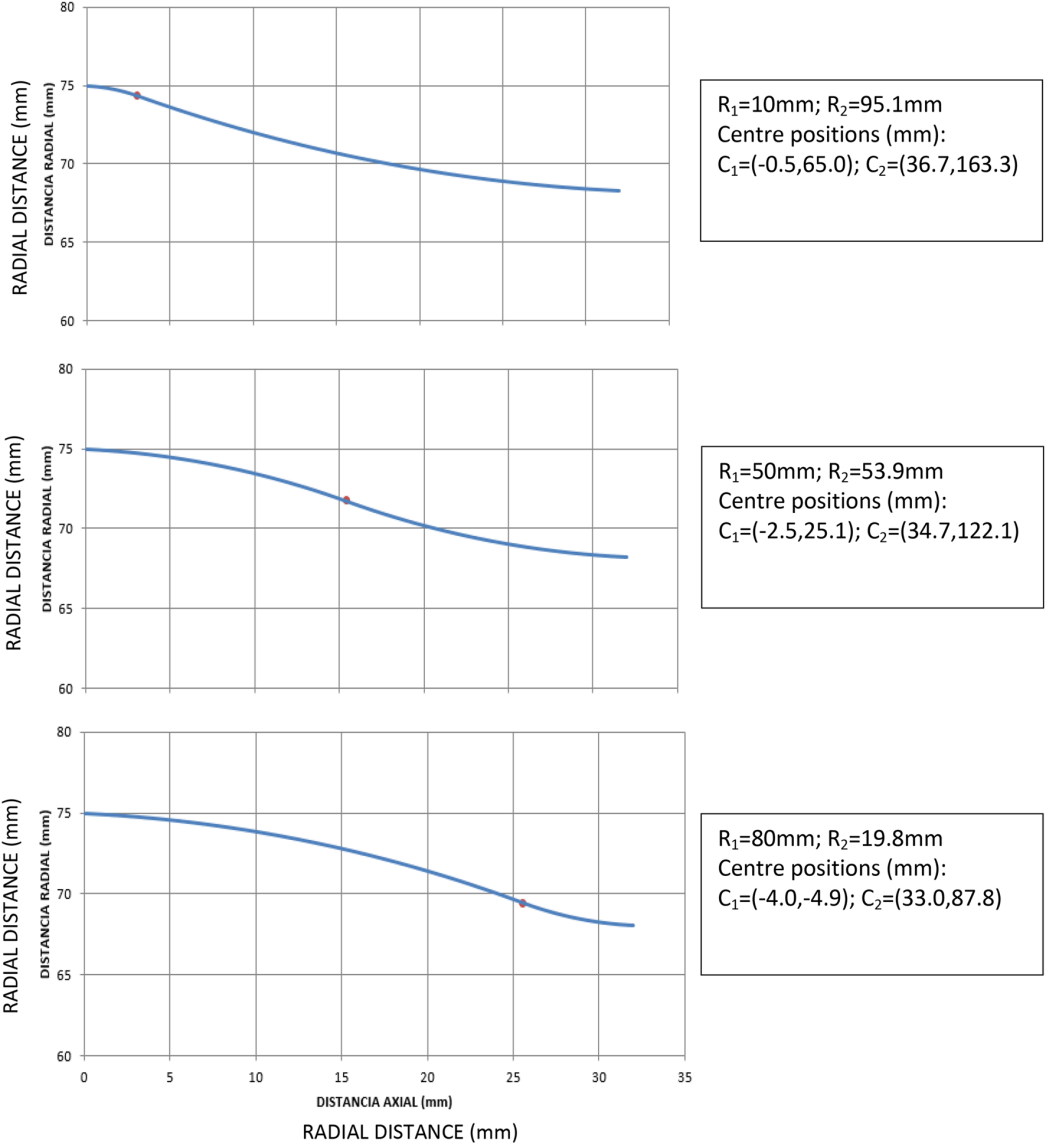
Roller profiles used in load transfer simulations at the double support phase.

To this end, 3 rollers of parameters *β* = 2.86°, a = 32 mm, e = 5 mm, R = 75 mm, s = 4 mm, were simulated for 3 different values of the radius of curvature of the convex arc, R_1_ = {10 mm, 50 mm, 80 mm}. The corresponding profiles are shown in Fig. 13, together with the calculated values of the second radius of curvature (concave arc), and the positions of the two centres of curvature, in Cartesian coordinates (x, y).

**Figure 14:**
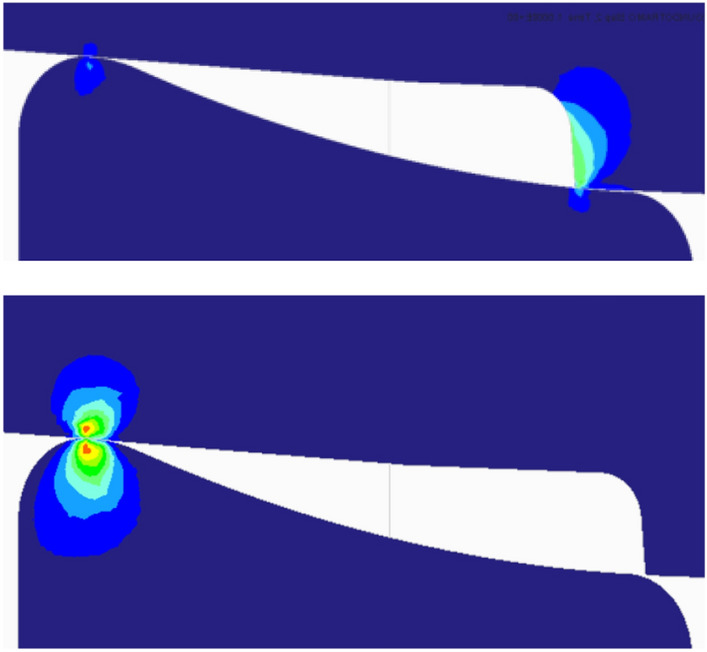
FEM graphs corresponding to the beginning (top) and end (bottom) of the double support phase.

In Fig. 14 the beginning and the end of the double contact phase are shown for the first roller (R_1_ = 10 mm, R_2_ = 95.1 mm), as obtained with a finite element software. Several static simulations have been performed between these two positions for each of the three profiles mentioned above.

**Figure 15:**
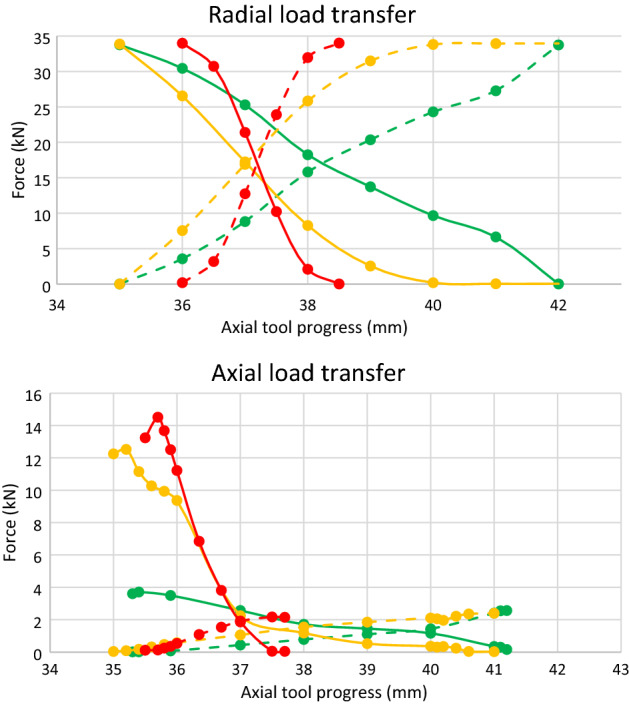
Radial and axial roller-wheel contact force transfer in the double-contact phase. Green: R_1_ = 10 mm; orange: R_1_ = 50 mm; Red: R_1_ = 80 mm. Dashed line: contact force in the convex zone of the roller (point A). Solid line: contact force between the machining edge and the concave zone of the roller (point B).

The most relevant results of the simulations are condensed in the graphs of Fig. 15. The green curves correspond to the roller with R_1_ = 10 mm; the curves in orange, to the roller with R_1_ = 50 mm; and the curves in red, to the roller with R_1_ = 80 mm. Solid lines are for (decreasing) contact forces at point B, whereas dashed lines are for (increasing) contact forces at point A (for identification of points A and B, see Figs. 2, 5, 7 or 12).

In view of these graphs, the conclusion is that the roller which concave section has greater radius is the one that produces a smoother, radial and axial load transfer. In general, the lower slope of the roller at point B, the smoother the load transfer will occur from support at point B to support at point A. This is achieved for small values of R_1_, but this radius cannot be arbitrarily small, since the contact pressure increases as it decreases. With a value of R_1_ = 10 mm, a Von Mises stress less than 4500 MPa has been obtained, which we consider a fairly acceptable value.

## Conclusions

In this article, a problem associated with wheel re-profiling process by under-floor lathes with a single cutting tool is first discussed. With these lathes an abrupt axial load transfer occurs when re-profiling, which is related with the profile geometry of the current support rollers used in the process.

Then, a new kind of support roller profile has been presented as a solution of the problem, being the concave-convex geometry its main feature. The methodology to design the parametrized optimum roller profile is developed and verified.

The essential difference between the process with the novel convex-concave-profile roller proposed in the article, and the conventional convex-profile roller, is shown in the lower part of Figs. 11 and 12, respectively. In fact, when the novel-profile roller is used, no axial load transfer occurs (Fig. 11), whereas currently used rollers lead to abrupt axial load transfer (Fig. 12).

Once the improvement of the convex-concave-profile rollers over ordinary convex-rollers has been revealed, an additional optimization was carried out on the former, using finite element static analysis along the process. Three profiles with different values of their concave-convex curvature radii were analysed. The results are presented in Sect. 4, where it is shown that the greater the radius of curvature of the concave section (or less that of the convex, the sum of both being fixed), the lower the load transfer in the double support. However, the radius of curvature of the convex section cannot be arbitrarily small, as it would lead to arbitrarily large values of the contact pressure, right where the roller supports most of the re-profiling process. For a roller 75 mm radius, its convex section radius of curvature of 10 mm is large enough to support a common wheel during its reprofiling process with existing lathes.

Novel-profile rollers, like those proposed in the article, have been implemented in an under-floor re-profiling lathe by a benchmark manufacturer that develop and supply advanced machine tools. This company has reported a significantly smoother behaviour of the process when using these novel rollers.

### Ethical approval

No part of this study was performed on any human or animal subjects.

## Abbreviations

*β*: Taper half-angle of the wheel
*γ*: Slope of the roller profile at the contact with the machining edge (variable as the re-profiling progresses)
*φ*: Angle between the roller-wheel contact force and its radial component, so that $\tan \varphi = \frac{{F_{A} }}{{F_{R} }}$
*a*: Roller axial length of its useful area.
C_1_ = (X_1_,Y_1_): Coordinates for the centre of curvature of the convex arc of the roller profile.
C_2_ = (X_2_,Y_2_): Coordinates for the centre of curvature of the concave arc of the roller profile.
*e*: Machining depth.
*F*_*A*_: Axial component of the roller-wheel contact force.
*F*_*R*_: Radial component of the roller-wheel contact force.
*R*: Roller radius at first contact with the wheel (point A).
*R*_1_ (*R*_2_): Radius of arc for the convex (concave) roller profile section.
*s*: Safety section length measured along the profile (the last section of the useful width)
${\mathrm{V}}_{\mathrm{a}}$: Approach velocity, or velocity with which the re-profiled surface approaches the roller
$${\mathrm{V}}_{\mathrm{m}}$$: Machining velocity.
*X*: Axial coordinate
*X*_*t*_: Axial coordinate corresponding to the point of tangency between convex and concave arches.
*Y*: Radial coordinate
